# Recent advances in understanding the role of lamins in health and disease

**DOI:** 10.12688/f1000research.9260.1

**Published:** 2016-10-19

**Authors:** Sita Reddy, Lucio Comai

**Affiliations:** 1 Department of Biochemistry and Molecular Biology, Institute for Genetic Medicine, Keck School of Medicine, University of Southern California, Los Angeles, CA, USA; 2 Department of Molecular Microbiology and Immunology, Institute for Genetic Medicine, Keck School of Medicine, University of Southern California, Los Angeles, CA, USA

**Keywords:** lamins, nuclear envelope, Hutchinson-Gilford progeria syndrome, Lamin Association Domains

## Abstract

Lamins are major components of the nuclear lamina, a network of proteins that supports the nuclear envelope in metazoan cells. Over the past decade, biochemical studies have provided support for the view that lamins are not passive bystanders providing mechanical stability to the nucleus but play an active role in the organization of the genome and the function of fundamental nuclear processes. It has also become apparent that lamins are critical for human health, as a large number of mutations identified in the gene that encodes for A-type lamins are associated with tissue-specific and systemic genetic diseases, including the accelerated aging disorder known as Hutchinson-Gilford progeria syndrome. Recent years have witnessed great advances in our understanding of the role of lamins in the nucleus and the functional consequences of disease-associated A-type lamin mutations. Many of these findings have been presented in comprehensive reviews. In this mini-review, we discuss recent breakthroughs in the role of lamins in health and disease and what lies ahead in lamin research.

## Lamins and the nuclear lamina

**Figure 1.**
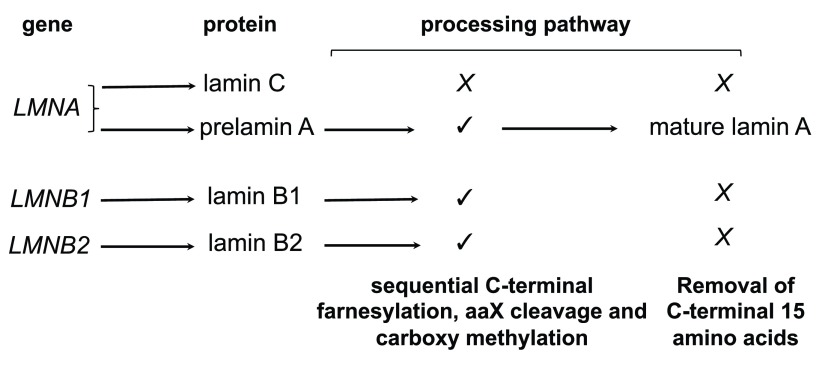
Major A-type and B-type lamins in mammals. Prelamin A, lamin B1, and lamin B2 contain a carboxy-terminal CaaX motif (CSIM in human prelamin A, CAIM in lamin B1, and CYVM in lamin B2; C is cysteine, S is serine, I is isoleucine, M is methionine, A is alanine, Y is tyrosine, and V is valine) which is modified by farnesylation. This is followed by proteolysis of the aaX residues and carboxy methylation at the C-terminal end of lamin A, B1, and B2. Prelamin A undergoes further processing to remove the carboxy-terminal 15 amino acids, including the farnesylated and carboxy methylated cysteine to generate mature lamin A. In Hutchinson-Gilford progeria syndrome cells, the second cleavage site in prelamin A is deleted, and this results in the accumulation of a permanently farnesylated and carboxy methylated prelamin A variant termed progerin. Terminal cleavage of prelamin A is catalyzed by the zinc metallopeptidase ZMPSTE24, an enzyme that has recently been implicated in clearing proteins through clogged endoplasmic reticulum translocon channel ^98^.

**Figure 2.**
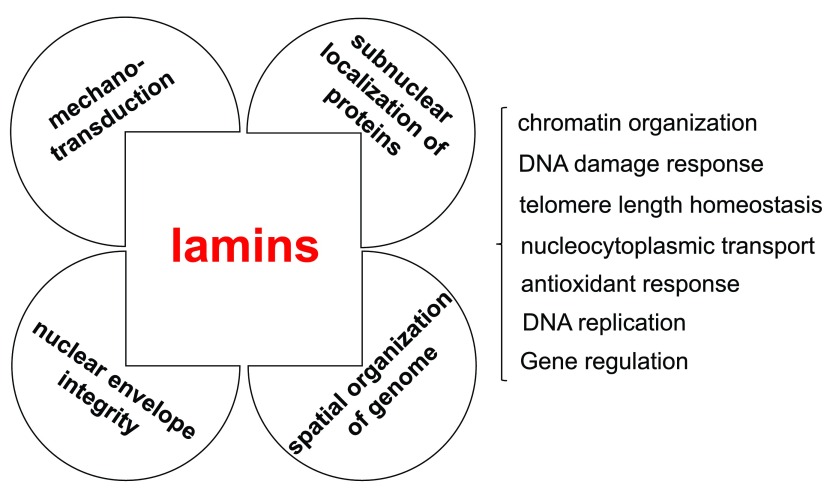
Lamins influence the mechanical properties of the nucleus and contribute to genome organization, function, and stability. Lamins have roles that support various aspects of nuclear structure and function. Lamins provide mechanical strength to the cell nucleus and contribute to cellular mechanotransduction. Lamins influence the nucleoplasmic environment and contribute to shaping the spatial organization of the genome. Lamins influence genome function and stability by contributing, through interactions with various nuclear factors, to the epigenetic regulation of chromatin, DNA replication and repair, and gene transcription.

Lamins are members of the family of intermediate filaments that are largely but not exclusively localized to the nuclear lamina, a multiprotein mesh structure found on the inner side of the nuclear membrane of most metazoan cells ^1– 4^. Mammalian cells have two types of lamins: A-type lamins, which are expressed in most terminally differentiated cells, and B-type lamins, which are expressed in most or all somatic cells ( Figure 1). A-type lamin A and C are encoded by the *LMNA* gene and generated by alternative splicing, whereas B-type lamin B1 and B2 are encoded by two separate genes: *LMNB1* and *LMNB2*. Short lamin C2 and lamin B3 isoforms encoded by the *LMNA* and *LMNB2*, respectively, are expressed only in gametes ^5, 6^. Two minor isoforms of lamin A (Δ10) and lamin C (C2) have also been identified, but their function and regulation are not yet fully understood ^3^. Lamin A, B1, and B2, but not lamin C, have a carboxy-terminal CaaX motif (C is cysteine, a is an aliphatic amino acid, and X is any amino acid) that undergoes sequential cysteine farnesylation, aaX cleavage, and carboxy methylation. Whereas these modifications are permanent on lamin B1 and B2, lamin A is synthesized as a prelamin A precursor that undergoes an additional processing step catalyzed by the Zn metallopeptidase STE24 (ZMPSTE24) that removes the carboxy-terminal 15-amino-acid tail, including the modified cysteine to generate mature lamin A. Farnesylation is thought to strengthen the association of B-type lamins with the inner nuclear membrane, while the lack of this modification in lamin A and C allows these lamins to be more loosely associated with the nuclear envelope and also occupy the nucleoplasmic space. Lamins are believed to provide a framework that supports the assembly and stability of the nuclear envelope and contributes to nuclear shape and mechanotransduction ^1, 2, 7, 8^. Moreover, a growing body of research has provided compelling evidence that lamins make significant contributions to the dynamic organization and function of the genome ^1– 4, 9^. Determining the function of lamins is of critical importance for human health because of the large number of mutations identified across the LMNA gene that are associated with a class of human disorders, collectively known as laminopathies, whose clinical symptoms include skeletal or cardiac muscular dystrophy, lipodystrophy, dysplasia, dermopathy, neuropathy, leukodystrophy, and accelerated aging ^9, 10^. The discovery in 2003 that Hutchinson-Gilford progeria syndrome (HGPS), a rare premature aging disease that affects children, is caused by a *de novo LMNA* mutation that leads to impaired processing of prelamin A and the production of a permanently farnesylated mutant lamin A protein termed progerin ^11, 12^ has led to an escalation in lamin research with the hope of finding a cure for this devastating disease. Expression of progerin causes severe cellular defects that affect nuclear morphology, chromatin organization, telomere length homeostasis, DNA repair, nucleoplasmic transport, and redox homeostasis ^13– 17^. Recent studies have provided critical information on the contribution of lamins to nuclear mechanics and the spatial organization of the nucleus ( Figure 2) and provided considerable experimental evidence for the hypothesis that lamin A mutations disrupt processes that are critical for nucleocytoplasmic mechanotransduction, nuclear positioning, chromatin organization and function, and responses to stress.

## Lamins in nuclear mechanobiology

The nucleus plays a critical role in the response to mechanical forces, and new research adds to a growing body of evidence implicating lamin A/C and the linker of nucleo-skeleton to cytoskeleton (LINC) complexes, which bridge the nuclear lamina to the cytoskeleton, in tissue adaptation to mechanical forces ^7, 8, 18^. Lamins form high-molecular-weight structures, and high-resolution microscopy data have revealed that A- and B-type lamins are organized in a distinct but interdependent meshwork of fibrils ^19^. Each of these structures is likely to contribute to maintaining the organization of the nuclear lamina and the shape of the nucleus. Yet the observation that depletion of lamin A/C increases nuclear deformability in response to mechanical stress suggests that lamin A/C fibrils play a prominent role in regulating the stiffness and elasticity of the nucleus ^20, 21^. Consistent with these data, differences in lamin A/C expression leading to changes in lamin A/C-to-B ratio have been demonstrated across distinct cell types, with higher lamin A/C levels observed in cells of tissues often subjected to mechanical torsion, including muscle and heart ^22^. Variations in the lamin A/C-to-lamin B ratio have also been observed during hematopoiesis ^23^, and it is likely that changes in lamin A/C expression affect nuclear stiffness in cancer cells, which may contribute to pathological outcomes, including metastasis ^24^. A recent study has also identified force-dependent changes in lamin A/C conformation ^25^, suggesting that other mechanisms of lamin A regulation contribute to adjusting nuclear shape in response to stress. Research on lamin A/C mutations linked to Emery-Dreifuss muscular dystrophy (EDMD) and dilated cardiomyopathy (DCM) further underscores a role of lamin A/C in nuclear mechanics ^26– 28^. These studies demonstrated that several disease-causing mutations compromise the stiffness of the nucleus and the integrity of the nuclear envelope, including the nuclear pore complex, in cells of the affected tissues. Remarkably, a recent report showed that muscle structure and function in an animal model of EDMD with tissue-specific alterations in nuclear mechanics are returned to normal by gene inactivation of the enzyme responsible for protein prenylation ^29^. Although the precise mechanism underlying this observation remains to be determined, it is possible that changes in the properties, physical interactions, or high-order structure formed by unfarnesylated lamin B confers protection against tissue-specific mechanical stress in this animal model. It is important to point out that not all *LMNA* gene mutations linked to EDMD or DMC, nor mutations associated with familial partial lipodystrophy, result in nuclear fragility ^27, 29^, suggesting that distinct mechanical properties or nuclear functions are affected by different lamin A mutations.

## Lamins in chromatin structure and spatial organization of the genome

Within the past few years, efforts have been directed at better understanding the relationship between lamins and genome organization and stability. Both A- and B-type lamins bind DNA *in vitro* ^30^ and associate with chromatin *in vivo* ^2, 31^, and their loss affects genome integrity ^32– 34^. Analysis of chromatin-lamin interactions using an *in vivo* tagging approach (DNA adenine methyltransferase identification, or DamID) ^35, 36^ demonstrated that lamins make dynamic contacts with large regions of chromatin, which have been termed lamina-associated domains (LADs), adjacent to the nuclear lamina. These domains are enriched in repressive histone markers, including dimethylated H3K9 and trimethylated H3K27, suggesting that LADs represent a repressive chromatin environment. In spite of these findings, the role of lamins in the formation of LAD remains unclear. A recent study has indicated that lamin C is sufficient for LAD formation at the nuclear lamina ^37^, and another has questioned the need of any lamin for the formation of these domains ^38^. Interestingly, whereas the DamID studies suggested a very high degree of concordance between lamin A/C- and lamin B-associated chromosome domains, recent work using a chromatin-immunoprecipitation approach has identified a subpopulation of lamin A/C that interacts with active regions of chromatin, in coordination with the lamin-associated factor LAP2α ^39^. These are likely interactions that occur within the nucleoplasmic space away from the nuclear lamina since LAP2α colocalizes with lamin A/C within the nuclear interior ^40, 41^. Importantly, both LAP2α levels and the nucleoplasmic pool of lamin A/C are dramatically reduced in the presence of the lamin A mutant progerin ^42, 43^, and these changes are thought to influence processes that are critical for cell proliferation. The conclusion of this and other recent studies on this topic is that a tight balance between lamin A/C and LAP2α must be maintained to ensure proper cell function, although how this is achieved remains to be worked out. Other studies have also demonstrated that lamins, together with other components of the nuclear lamina termed nuclear envelope transmembrane proteins (NETs), contribute to tissue-specific organization of the genome and influence gene expression by securing peripheral heterochromatin to the nuclear lamina and repositioning genes within the nucleus during cell differentiation ^44, 45^. The NET lamin B receptor (LBR) has also been recently implicated in the recruitment of the X chromosome to the nuclear lamina to promote X-inactive-specific transcript (Xist)-mediated gene silencing ^46^. Taken together with the observation that muscle-specific chromatin reorganization is disrupted in an animal model of EDMD ^28^, these findings suggest that altered spatial organization of heterochromatin or incorrect positioning of genes contributes to the development of tissue-specific pathologies in at least a subset of the diseases that have been linked to mutations in lamins or NETs.

Lamin A and the mutant progerin have been shown to differentially influence the stability and spatial localization of epigenetic regulators of chromatin structure ^31, 47^, and several studies have reported a gradual decrease in peripheral heterochromatin and global loss of several histone markers of heterochromatin in progerin-expressing cells ^48– 52^. However, a recent study has added a twist to this story by showing that increased levels of the heterochromatic histone modification trimethyl H3K9 contribute to the development of the progeroid phenotype ^53^. The authors demonstrated a direct interaction between lamin A and SUV39h1, a chromatin modifier that is responsible for H3K9me3. Progerin also binds SUV39h1, albeit more tightly than lamin A, which results in increased levels of H3K9me3 in progeria cells. This is an unanticipated result that differs from other studies. A clarification of the type of epigenetic changes caused by progerin requires further investigation, but it is possible, as suggested by the authors of this study, that the decreased heterochromatinization reported by others reflects an *in vitro* cell passage-dependent effect rather than an *in vivo* process. The concept that progerin disrupts lamin A-protein interactions that locally influence chromatin organization is supported by another recent study ^54^. In this work, lamin A is shown to recruit chromatin modifiers through interactions with barrier-to-autointegration factor (BAF), a family of proteins that are thought to mediate interactions between various factors and chromatin ^55^. As seen with SUV39h1, progerin binds stronger than lamin A to BAF and this interaction results in BAF mislocalization, leading to epigenetic changes that alter chromosome organization and are likely to contribute to cell dysfunction.

## Lamins in the regulation of nuclear processes

Fundamental nuclear processes such as transcription, replication, and DNA repair are tightly connected to the spatial organization of the genome and their function relies on the timely recruitment of specific factors to the proper chromosome locations. Recent studies have suggested that progerin disrupts these processes by preventing the recruitment of specific factors to their target site. One example is sirtuin 6 (SIRT6), a protein involved in multiple processes related to genomic stability, stress resistance, telomere maintenance, and energy homeostasis ^56^. A study has shown that both lamin A and progerin bind SIRT6, but a stronger interaction with progerin results in SIRT6 sequestration to the nuclear lamina, which prevents SIRT6 from relocalizing to sites of DNA damage. Taken together with prior data showing that progerin affects the function of other DNA repair factors ^16^, these results underscore the significant hurdle imposed by this mutant lamin A on the pathways that maintain genome integrity. Intriguingly, SIRT6 also plays a role in the recruitment to telomeres of the Werner syndrome protein (WRN) ^57^, a protein whose loss-of-function mutations cause genetic instability leading to an adult-onset type of progeria ^58^. Although it is not known whether WRN function is affected in cells expressing progerin, it is possible that mislocalization of SIRT6 prevents WRN recruitment to telomeres, and this may contribute to telomere dysfunction in HGPS cells. Unfortunately, overexpression of SIRT6 is not sufficient to rescue progeria cell dysfunction, thus limiting the usefulness of potential SIRT6-based therapeutic interventions ^59^.

In support of the idea that sequestration by progerin is a major mechanism leading to cell dysfunction, it has recently been reported that progerin binds NRF2, a transcription factor that regulates the expression of genes involved in maintaining redox homeostasis ^60^, and relocates it to the nuclear lamina ^61^. Oxidative stress, which has been linked to defective nucleocytoplasmic transport and is likely contributing to persistent DNA damage in HGPS cells ^62– 66^, appears to be a central factor in the pathophysiology of progeria. Since ectopic expression of constitutively active NRF2 ameliorates several of the cellular defects of progeria cells, deregulation of NRF2 function has been suggested to be a primary driver of accelerated aging. Although it is unclear how constitutively active NRF2 escapes sequestration to the nuclear lamina by progerin, these findings suggest that therapeutic approaches that restore NRF2 function may be beneficial to patients with HGPS. Deregulation of NRF2 has also been observed in cells from muscular dystrophy patients expressing certain missense lamin A mutants that tend to mislocalize to the cytoplasm ^67^. However, this study reported activation rather than repression of NRF2 in these cells through a mechanism that does not involve lamin A binding.

## Therapeutic approaches to Hutchinson-Gilford progeria syndrome

Translation of basic science findings into therapeutic approaches is the uttermost goal of biomedical research. In this regard, the Progeria Research Foundation ( http://www.progeriaresearch.org), a non-profit organization founded by the parents of a child with HGPS, has been influential in raising awareness and funds for research on finding a cure for this disease, and these efforts have contributed significantly to the large increase in lamin A research during the last decade. The cellular toxicity of partially processed prelamin A mutants like progerin is due primarily to the presence of the farnesyl group at the carboxy-terminal cysteine. Drugs that inhibit protein farnesyl transferase (farnesyl transferase inhibitors, or FTIs) have been shown to improve the cellular phenotype of progeria cells and ameliorate the pathology of mouse models of the disease ^68– 79^. FTIs may also hold therapeutic potential for patients carrying EDMD-linked mutations ^29^. Driven by these findings, the Progeria Research Foundation sponsored a single-arm clinical trial using the FTI lonafarnib and reported improvements in weight gain, bone structure, and the cardiovascular system of patients with progeria ^80^. However, FTIs are far from being a cure for progeria and better drugs are urgently needed. Since then, a new clinical trial using pharmacological inhibitors of the mevalonate biosynthetic pathway (pravastatin, zoledronic acid, and lonafarnib) has been under way, and preliminary findings have just been published ^81^. They indicate that even though the three-drug regimen improves bone size and mineral density, no additional benefit over the one-drug treatment is observed in cardiovascular structure and function. Small molecules that reduce the accumulation of progerin (that is, rapamycin) or influence the microtubule network (that is, remodelin) have recently been shown to have beneficial effects in tissue culture models of progeria ^82– 84^, and they offer new opportunities for therapeutic intervention. Rapamycin may have a therapeutic effect on other laminopathies, since temsirolimus, a rapamycin analog, has been shown to counteract the deterioration of cardiac function in a murine model of cardiomyopathy caused by a lamin A mutation ^85^. Future studies in animal models will be crucial to better understand the efficacy and usefulness of these and other new drugs in treating patients with *LMNA* mutations.

## Future challenges

The number of articles published on lamins has grown exponentially during the last few years, and tremendous progress has been made in understanding the biological properties of these proteins and lamin A mutants associated with disease. In spite of this gained knowledge, a number of challenges remain. More studies are needed to better understand the relative contributions of lamin A and lamin C to the dynamic spatial organization of the genome in different cell types during development and differentiation. The potential role of lamins in organizing transcription or replication units and DNA damage repair foci needs to be further explored, and future investigations are expected to provide important insights on these topics. Relatively little is known about the molecular mechanisms of tissue-specific disorders caused by *LMNA* missense mutations that do not affect prelamin A processing. A study in cells from a mouse model of DCM has recently shown that expression of a missense mutant N195K-lamin A (N195K) impairs nucleocytoplasmic shuttling of a key factor in cardiac development ^86^. These results suggest that a single amino acid change in the lamin A polypeptide induces structural alterations that influence the intracellular distribution and function of a cell-type-specific factor. In a new report, two missense *LMNA* mutations linked to muscular dystrophy (R453W and R482W) have been shown to disrupt LAD and alter heterochromatin organization during myogenic differentiation ^87^. These findings strengthen the idea that lamin A/C contributes to the spatial and structural remodeling of chromatin that takes place during cell differentiation. There are hundreds of mutations in the *LMNA* gene known to be associated with tissue-specific diseases ^9^. Thus, one may speculate that at least some of these mutations cause tissue-specific defects by affecting the localization or subcellular distribution of factors that, by regulating cell-type-specific regulatory genes or pathways, orchestrate the spatial organization and function of the nucleus. There is also more to learn about the functions of lamin B1 and B2, which, in spite of the high degree of sequence conservation, do not seem to be functionally redundant ^88, 89^. There is strong evidence that B-type lamins are required for DNA replication, and recent work has identified a specific role for lamin B1 during the elongation phase of this process ^32, 90– 92^. Both lamin B1 and B2 have also been implicated in neuronal migration and survival, and altered distribution of the nuclear pore complex has been observed in lamin B1-deficient cortical neurons ^93– 97^. This defect has been suggested to affect nucleocytoplasmic shuttling of certain factors ^97^, which is reminiscent of the cellular defect caused by the lamin A mutation associated with DCM discussed above ^86^. These are findings that bring excitement as well as challenges to an area of research that is predicted to expand further over the next several years.

## Abbreviations

BAF, barrier-to-autointegration factor; DamID, DNA adenine methyltransferase identification; DCM, dilated cardiomyopathy; EDMD, Emery-Dreifuss muscular dystrophy; FTI, farnesyl transferase inhibitor; HGPS, Hutchinson-Gilford progeria syndrome; LAD, lamina-associated domain; LAP2α, lamin-associated protein 2α; NET, nuclear envelope transmembrane protein; SIRT6, sirtuin 6; WRN, Werner syndrome protein.

## References

[1] Holmer L Worman HJ: Inner nuclear membrane proteins: functions and targeting. *Cell Mol Life Sci.* 2001;58(12–13):1741–7. 10.1007/PL00000813 11766875 PMC11337314

[2] Goldman RD Gruenbaum Y Moir RD: Nuclear lamins: building blocks of nuclear architecture. *Genes Dev.* 2002;16(5):533–47. 10.1101/gad.960502 11877373

[3] Gruenbaum Y Foisner R: Lamins: nuclear intermediate filament proteins with fundamental functions in nuclear mechanics and genome regulation. *Annu Rev Biochem.* 2015;84:131–64. 10.1146/annurev-biochem-060614-034115 25747401

[4] Burke B Stewart CL: The nuclear lamins: flexibility in function. *Nat Rev Mol Cell Biol.* 2013;14(1):13–24. 10.1038/nrm3488 23212477

[5] Furukawa K Hotta Y: cDNA cloning of a germ cell specific lamin B3 from mouse spermatocytes and analysis of its function by ectopic expression in somatic cells. *EMBO J.* 1993;12(1):97–106. 8094052 10.1002/j.1460-2075.1993.tb05635.x PMC413179

[6] Furukawa K Inagaki H Hotta Y: Identification and cloning of an mRNA coding for a germ cell-specific A-type lamin in mice. *Exp Cell Res.* 1994;212(2):426–30. 10.1006/excr.1994.1164 8187835

[7] Osmanagic-Myers S Dechat T Foisner R: Lamins at the crossroads of mechanosignaling. *Genes Dev.* 2015;29(3):225–37. 10.1101/gad.255968.114 25644599 PMC4318140

[8] Davidson PM Lammerding J: Broken nuclei--lamins, nuclear mechanics, and disease. *Trends Cell Biol.* 2014;24(4):247–56. 10.1016/j.tcb.2013.11.004 24309562 PMC3972295

[9] Worman HJ: Nuclear lamins and laminopathies. *J Pathol.* 2012;226(2):316–25. 10.1002/path.2999 21953297 PMC6673656

[10] Broers JL Hutchison CJ Ramaekers FC: Laminopathies. *J Pathol.* 2004;204(4):478–88. 10.1002/path.1655 15495262

[11] De Sandre-Giovannoli A Bernard R Cau P: Lamin a truncation in Hutchinson-Gilford progeria. *Science.* 2003;300(5628):2055. 10.1126/science.1084125 12702809

[12] Eriksson M Brown WT Gordon LB: Recurrent *de novo* point mutations in lamin A cause Hutchinson-Gilford progeria syndrome. *Nature.* 2003;423(6937):293–8. 10.1038/nature01629 12714972 PMC10540076

[13] Gonzalo S Eissenberg JC: Tying up loose ends: telomeres, genomic instability and lamins. *Curr Opin Genet Dev.* 2016;37:109–18. 10.1016/j.gde.2016.03.003 27010504 PMC4914467

[14] Capell BC Collins FS: Human laminopathies: nuclei gone genetically awry. *Nat Rev Genet.* 2006;7(12):940–52. 10.1038/nrg1906 17139325

[15] Ghosh S Zhou Z: Genetics of aging, progeria and lamin disorders. *Curr Opin Genet Dev.* 2014;26:41–6. 10.1016/j.gde.2014.05.003 25005744

[16] Gonzalo S Kreienkamp R: DNA repair defects and genome instability in Hutchinson-Gilford Progeria Syndrome. *Curr Opin Cell Biol.* 2015;34:75–83. 10.1016/j.ceb.2015.05.007 26079711 PMC4522337

[17] Reddy S Comai L: Lamin A, farnesylation and aging. *Exp Cell Res.* 2012;318(1):1–7. 10.1016/j.yexcr.2011.08.009 21871450 PMC4209918

[18] Graham DM Burridge K: Mechanotransduction and nuclear function. *Curr Opin Cell Biol.* 2016;40:98–105. 10.1016/j.ceb.2016.03.006 27018929 PMC4887340

[19] Shimi T Kittisopikul M Tran J: Structural organization of nuclear lamins,A, C, B1, and B2 revealed by superresolution microscopy. *Mol Biol Cell.* 2015;26(22):4075–86. 10.1091/mbc.E15-07-0461 26310440 PMC4710238

[20] Pajerowski JD Dahl KN Zhong FL: Physical plasticity of the nucleus in stem cell differentiation. *Proc Natl Acad Sci U S A.* 2007;104(40):15619–24. 10.1073/pnas.0702576104 17893336 PMC2000408

[21] Guilluy C Osborne LD van Landeghem L: Isolated nuclei adapt to force and reveal a mechanotransduction pathway in the nucleus. *Nat Cell Biol.* 2014;16(4):376–81. 10.1038/ncb2927 24609268 PMC4085695

[22] Swift J Ivanovska IL Buxboim A: Nuclear lamin-A scales with tissue stiffness and enhances matrix-directed differentiation. *Science.* 2013;341(6149):1240104. 10.1126/science.1240104 23990565 PMC3976548

[23] Shin JW Spinler KR Swift J: Lamins regulate cell trafficking and lineage maturation of adult human hematopoietic cells. *Proc Natl Acad Sci U S A.* 2013;110(47):18892–7. 10.1073/pnas.1304996110 24191023 PMC3839750

[24] Bell ES Lammerding J: Causes and consequences of nuclear envelope alterations in tumour progression. *Eur J Cell Biol.* 2016; pii: S0171-9335(16)30109-1. 10.1016/j.ejcb.2016.06.007 27397692 PMC5110382

[25] Ihalainen TO Aires L Herzog FA: Differential basal-to-apical accessibility of lamin A/C epitopes in the nuclear lamina regulated by changes in cytoskeletal tension. *Nat Mater.* 2015;14(12):1252–61. 10.1038/nmat4389 26301768 PMC4655446

[26] Dialynas G Flannery KM Zirbel LN: *LMNA* variants cause cytoplasmic distribution of nuclear pore proteins in *Drosophila* and human muscle. *Hum Mol Genet.* 2012;21(7):1544–56. 10.1093/hmg/ddr592 22186027 PMC3298278

[27] Zwerger M Jaalouk DE Lombardi ML: Myopathic lamin mutations impair nuclear stability in cells and tissue and disrupt nucleo-cytoskeletal coupling. *Hum Mol Genet.* 2013;22(12):2335–49. 10.1093/hmg/ddt079 23427149 PMC3658163

[28] Mattout A Pike BL Towbin BD: An EDMD mutation in C. elegans lamin blocks muscle-specific gene relocation and compromises muscle integrity. *Curr Biol.* 2011;21(19):1603–14. 10.1016/j.cub.2011.08.030 21962710

[29] Zuela N Zwerger M Levin T: Impaired mechanical response of an EDMD mutation leads to motility phenotypes that are repaired by loss of prenylation. *J Cell Sci.* 2016;129(29):1781–91. 10.1242/jcs.184309 27034135

[30] Shoeman RL Traub P: The *in vitro* DNA-binding properties of purified nuclear lamin proteins and vimentin. *J Biol Chem.* 1990;265(16):9055–61. 2345165

[31] Han X Feng X Rattner JB: Tethering by lamin A stabilizes and targets the ING1 tumour suppressor. *Nat Cell Biol.* 2008;10(11):1333–40. 10.1038/ncb1792 18836436

[32] Butin-Israeli V Adam SA Jain N: Role of lamin b1 in chromatin instability. *Mol Cell Biol.* 2015;35(5):884–98. 10.1128/MCB.01145-14 25535332 PMC4323489

[33] Gonzalo S: DNA damage and lamins. *Adv Exp Med Biol.* 2014;773:377–99. 10.1007/978-1-4899-8032-8_17 24563357 PMC4081481

[34] Camps J Erdos MR Ried T: The role of lamin B1 for the maintenance of nuclear structure and function. *Nucleus.* 2015;6(1):8–14. 10.1080/19491034.2014.1003510 25602590 PMC4615282

[35] Guelen L Pagie L Brasset E: Domain organization of human chromosomes revealed by mapping of nuclear lamina interactions. *Nature.* 2008;453(7197):948–51. 10.1038/nature06947 18463634

[36] Meuleman W Peric-Hupkes D Kind J: Constitutive nuclear lamina-genome interactions are highly conserved and associated with A/T-rich sequence. *Genome Res.* 2013;23(2):270–80. 10.1101/gr.141028.112 23124521 PMC3561868

[37] Harr JC Luperchio TR Wong X: Directed targeting of chromatin to the nuclear lamina is mediated by chromatin state and A-type lamins. *J Cell Biol.* 2015;208(1):33–52. 10.1083/jcb.201405110 25559185 PMC4284222

[38] Amendola M van Steensel B: Nuclear lamins are not required for lamina-associated domain organization in mouse embryonic stem cells. *EMBO Rep.* 2015;16(5):610–7. 10.15252/embr.201439789 25784758 PMC4428043

[39] Gesson K Rescheneder P Skoruppa MP: A-type lamins bind both hetero- and euchromatin, the latter being regulated by lamina-associated polypeptide 2 alpha. *Genome Res.* 2016;26(4):462–73. 10.1101/gr.196220.115 26798136 PMC4817770

[40] Naetar N Korbei B Kozlov S: Loss of nucleoplasmic LAP2alpha-lamin A complexes causes erythroid and epidermal progenitor hyperproliferation. *Nat Cell Biol.* 2008;10(11):1341–8. 10.1038/ncb1793 18849980

[41] Dechat T Korbei B Vaughan OA: Lamina-associated polypeptide 2alpha binds intranuclear A-type lamins. *J Cell Sci.* 2000;113(Pt 19):3473–84. 10984438 10.1242/jcs.113.19.3473

[42] Chojnowski A Ong PF Wong ES: Progerin reduces LAP2α-telomere association in Hutchinson-Gilford progeria. *eLife.* 2015;4: e07759. 10.7554/eLife.07759 26312502 PMC4565980

[43] Vidak S Kubben N Dechat T: Proliferation of progeria cells is enhanced by lamina-associated polypeptide 2α (LAP2α) through expression of extracellular matrix proteins. *Genes Dev.* 2015;29(19):2022–36. 10.1101/gad.263939.115 26443848 PMC4604344

[44] Robson MI de Las Heras JI Czapiewski R: Tissue-Specific Gene Repositioning by Muscle Nuclear Membrane Proteins Enhances Repression of Critical Developmental Genes during Myogenesis. *Mol Cell.* 2016;62(6):834–47. 10.1016/j.molcel.2016.04.035 27264872 PMC4914829

[45] Solovei I Wang AS Thanisch K: LBR and lamin A/C sequentially tether peripheral heterochromatin and inversely regulate differentiation. *Cell.* 2013;152(3):584–98. 10.1016/j.cell.2013.01.009 23374351

[46] Chen CK Blanco M Jackson C: Xist recruits the X chromosome to the nuclear lamina to enable chromosome-wide silencing. *Science.* 2016; pii: aae0047. 10.1126/science.aae0047 27492478

[47] Pegoraro G Kubben N Wickert U: Ageing-related chromatin defects through loss of the NURD complex. *Nat Cell Biol.* 2009;11(10):1261–7. 10.1038/ncb1971 19734887 PMC2779731

[48] Columbaro M Capanni C Mattioli E: Rescue of heterochromatin organization in Hutchinson-Gilford progeria by drug treatment. *Cell Mol Life Sci.* 2005;62(22):2669–78. 10.1007/s00018-005-5318-6 16261260 PMC2773834

[49] Goldman RD Shumaker DK Erdos MR: Accumulation of mutant lamin A causes progressive changes in nuclear architecture in Hutchinson-Gilford progeria syndrome. *Proc Natl Acad Sci U S A.* 2004;101(24):8963–8. 10.1073/pnas.0402943101 15184648 PMC428455

[50] McCord RP Nazario-Toole A Zhang H: Correlated alterations in genome organization, histone methylation, and DNA-lamin A/C interactions in Hutchinson-Gilford progeria syndrome. *Genome Res.* 2013;23(2):260–9. 10.1101/gr.138032.112 23152449 PMC3561867

[51] Scaffidi P Misteli T: Reversal of the cellular phenotype in the premature aging disease Hutchinson-Gilford progeria syndrome. *Nat Med.* 2005;11(4):440–5. 10.1038/nm1204 15750600 PMC1351119

[52] Shumaker DK Dechat T Kohlmaier A: Mutant nuclear lamin A leads to progressive alterations of epigenetic control in premature aging. *Proc Natl Acad Sci U S A.* 2006;103(23):8703–8. 10.1073/pnas.0602569103 16738054 PMC1472659

[53] Liu B Wang Z Zhang L: Depleting the methyltransferase Suv39h1 improves DNA repair and extends lifespan in a progeria mouse model. *Nat Commun.* 2013;4:1868. 10.1038/ncomms2885 23695662 PMC3674265

[54] Loi M Cenni V Duchi S: Barrier-to-autointegration factor (BAF) involvement in prelamin A-related chromatin organization changes. *Oncotarget.* 2016;7(13):15662–77. 10.18632/oncotarget.6697 26701887 PMC4941268

[55] Jamin A Wiebe MS: Barrier to Autointegration Factor (BANF1): interwoven roles in nuclear structure, genome integrity, innate immunity, stress responses and progeria. *Curr Opin Cell Biol.* 2015;34:61–8. 10.1016/j.ceb.2015.05.006 26072104 PMC4522355

[56] Kugel S Mostoslavsky R: Chromatin and beyond: the multitasking roles for SIRT6. *Trends Biochem Sci.* 2014;39(2):72–81. 10.1016/j.tibs.2013.12.002 24438746 PMC3912268

[57] Michishita E McCord RA Berber E: SIRT6 is a histone H3 lysine 9 deacetylase that modulates telomeric chromatin. *Nature.* 2008;452(7186):492–6. 10.1038/nature06736 18337721 PMC2646112

[58] Li B Jog S Candelario J: Altered nuclear functions in progeroid syndromes: a paradigm for aging research. *ScientificWorldJournal.* 2009;9:1449–62. 10.1100/tsw.2009.159 20024518 PMC4213125

[59] Ghosh S Liu B Wang Y: Lamin A Is an Endogenous SIRT6 Activator and Promotes SIRT6-Mediated DNA Repair. *Cell Rep.* 2015;13(7):1396–406. 10.1016/j.celrep.2015.10.006 26549451

[60] Ma Q: Role of nrf2 in oxidative stress and toxicity. *Annu Rev Pharmacol Toxicol.* 2013;53:401–26. 10.1146/annurev-pharmtox-011112-140320 23294312 PMC4680839

[61] Kubben N Zhang W Wang L: Repression of the Antioxidant NRF2 Pathway in Premature Aging. *Cell.* 2016;165(6):1361–74. 10.1016/j.cell.2016.05.017 27259148 PMC4893198

[62] Datta S Snow CJ Paschal BM: A pathway linking oxidative stress and the Ran GTPase system in progeria. *Mol Biol Cell.* 2014;25(8):1202–15. 10.1091/mbc.E13-07-0430 24523287 PMC3982987

[63] Richards SA Muter J Ritchie P: The accumulation of un-repairable DNA damage in laminopathy progeria fibroblasts is caused by ROS generation and is prevented by treatment with N-acetyl cysteine. *Hum Mol Genet.* 2011;20(20):3997–4004. 10.1093/hmg/ddr327 21807766

[64] Lattanzi G Marmiroli S Facchini A: Nuclear damages and oxidative stress: new perspectives for laminopathies. *Eur J Histochem.* 2012;56(4):e45. 10.4081/ejh.2012.e45 23361241 PMC3567764

[65] Sieprath T Corne TD Nooteboom M: Sustained accumulation of prelamin A and depletion of lamin A/C both cause oxidative stress and mitochondrial dysfunction but induce different cell fates. *Nucleus.* 2015;6(3):236–46. 10.1080/19491034.2015.1050568 25996284 PMC4615646

[66] Sieprath T Darwiche R De Vos WH: Lamins as mediators of oxidative stress. *Biochem Biophys Res Commun.* 2012;421(4):635–9. 10.1016/j.bbrc.2012.04.058 22538370

[67] Dialynas G Shrestha OK Ponce JM: Myopathic lamin mutations cause reductive stress and activate the nrf2/keap-1 pathway. *PLoS Genet.* 2015;11(5):e1005231. 10.1371/journal.pgen.1005231 25996830 PMC4440730

[68] Candelario J Borrego S Reddy S: Accumulation of distinct prelamin A variants in human diploid fibroblasts differentially affects cell homeostasis. *Exp Cell Res.* 2011;317(3):319–29. 10.1016/j.yexcr.2010.10.014 20974128

[69] Candelario J Sudhakar S Navarro S: Perturbation of wild-type lamin A metabolism results in a progeroid phenotype. *Aging Cell.* 2008;7(3):355–67. 10.1111/j.1474-9726.2008.00393.x 18363904 PMC2527236

[70] Lattanzi G: Prelamin A-mediated nuclear envelope dynamics in normal and laminopathic cells. *Biochem Soc Trans.* 2011;39(6):1698–704. 10.1042/BST20110657 22103510

[71] Yang SH Chang SY Andres DA: Assessing the efficacy of protein farnesyltransferase inhibitors in mouse models of progeria. *J Lipid Res.* 2010;51(2):400–5. 10.1194/jlr.M002808 19965595 PMC2803242

[72] Capell BC Erdos MR Madigan JP: Inhibiting farnesylation of progerin prevents the characteristic nuclear blebbing of Hutchinson-Gilford progeria syndrome. *Proc Natl Acad Sci U S A.* 2005;102(36):12879–84. 10.1073/pnas.0506001102 16129833 PMC1200293

[73] Capell BC Olive M Erdos MR: A farnesyltransferase inhibitor prevents both the onset and late progression of cardiovascular disease in a progeria mouse model. *Proc Natl Acad Sci U S A.* 2008;105(41):15902–7. 10.1073/pnas.0807840105 18838683 PMC2562418

[74] Fong LG Frost D Meta M: A protein farnesyltransferase inhibitor ameliorates disease in a mouse model of progeria. *Science.* 2006;311(5767):1621–3. 10.1126/science.1124875 16484451

[75] Glynn MW Glover TW: Incomplete processing of mutant lamin A in Hutchinson-Gilford progeria leads to nuclear abnormalities, which are reversed by farnesyltransferase inhibition. *Hum Mol Genet.* 2005;14(20):2959–69. 10.1093/hmg/ddi326 16126733

[76] Toth JI Yang SH Qiao X: Blocking protein farnesyltransferase improves nuclear shape in fibroblasts from humans with progeroid syndromes. *Proc Natl Acad Sci U S A.* 2005;102(36):12873–8. 10.1073/pnas.0505767102 16129834 PMC1193538

[77] Yang SH Meta M Qiao X: A farnesyltransferase inhibitor improves disease phenotypes in mice with a Hutchinson-Gilford progeria syndrome mutation. *J Clin Invest.* 2006;116(8):2115–21. 10.1172/JCI28968 16862216 PMC1513052

[78] Mallampalli MP Huyer G Bendale P: Inhibiting farnesylation reverses the nuclear morphology defect in a HeLa cell model for Hutchinson-Gilford progeria syndrome. *Proc Natl Acad Sci U S A.* 2005;102(40):14416–21. 10.1073/pnas.0503712102 16186497 PMC1242289

[79] Yang SH Qiao X Fong LG: Treatment with a farnesyltransferase inhibitor improves survival in mice with a Hutchinson-Gilford progeria syndrome mutation. *Biochim Biophys Acta.* 2008;1781(1–2):36–9. 10.1016/j.bbalip.2007.11.003 18082640 PMC2266774

[80] Gordon LB Kleinman ME Miller DT: Clinical trial of a farnesyltransferase inhibitor in children with Hutchinson-Gilford progeria syndrome. *Proc Natl Acad Sci U S A.* 2012;109(41):16666–71. 10.1073/pnas.1202529109 23012407 PMC3478615

[81] Gordon LB Kleinman ME Massaro J: Clinical Trial of the Protein Farnesylation Inhibitors Lonafarnib, Pravastatin, and Zoledronic Acid in Children With Hutchinson-Gilford Progeria Syndrome. *Circulation.* 2016;134(2):114–25. 10.1161/circulationaha.116.022188 27400896 PMC4943677

[82] Cao K Graziotto JJ Blair CD: Rapamycin reverses cellular phenotypes and enhances mutant protein clearance in Hutchinson-Gilford progeria syndrome cells. *Sci Transl Med.* 2011;3(89):89ra58. 10.1126/scitranslmed.3002346 21715679

[83] Cenni V Capanni C Columbaro M: Autophagic degradation of farnesylated prelamin A as a therapeutic approach to lamin-linked progeria. *Eur J Histochem.* 2011;55(4):e36. 10.4081/ejh.2011.e36 22297442 PMC3284238

[84] Larrieu D Britton S Demir M: Chemical inhibition of NAT10 corrects defects of laminopathic cells. *Science.* 2014;344(6183):527–32. 10.1126/science.1252651 24786082 PMC4246063

[85] Choi JC Muchir A Wu W: Temsirolimus activates autophagy and ameliorates cardiomyopathy caused by lamin A/C gene mutation. *Sci Transl Med.* 2012;4(144):144ra102. 10.1126/scitranslmed.3003875 22837537 PMC3700376

[86] Ho CY Jaalouk DE Vartiainen MK: Lamin A/C and emerin regulate MKL1-SRF activity by modulating actin dynamics. *Nature.* 2013;497(7450):507–11. 10.1038/nature12105 23644458 PMC3666313

[87] Perovanovic J Dell'Orso S Gnochi VF: Laminopathies disrupt epigenomic developmental programs and cell fate. *Sci Transl Med.* 2016;8(335):335ra58. 10.1126/scitranslmed.aad4991 27099177 PMC4939618

[88] Lee JM Tu Y Tatar A: Reciprocal knock-in mice to investigate the functional redundancy of lamin B1 and lamin B2. *Mol Biol Cell.* 2014;25(10):1666–75. 10.1091/mbc.E14-01-0683 24672053 PMC4019497

[89] Hutchison CJ: B-type lamins in health and disease. *Semin Cell Dev Biol.* 2014;29:158–63. 10.1016/j.semcdb.2013.12.012 24380701 PMC4053831

[90] Moir RD Montag-Lowy M Goldman RD: Dynamic properties of nuclear lamins: lamin B is associated with sites of DNA replication. *J Cell Biol.* 1994;125(6):1201–12. 10.1083/jcb.125.6.1201 7911470 PMC2290916

[91] Moir RD Spann TP Herrmann H: Disruption of nuclear lamin organization blocks the elongation phase of DNA replication. *J Cell Biol.* 2000;149(6):1179–92. 10.1083/jcb.149.6.1179 10851016 PMC2175110

[92] Spann TP Moir RD Goldman AE: Disruption of nuclear lamin organization alters the distribution of replication factors and inhibits DNA synthesis. *J Cell Biol.* 1997;136(6):1201–12. 10.1083/jcb.136.6.1201 9087437 PMC2132512

[93] Coffinier C Chang SY Nobumori C: Abnormal development of the cerebral cortex and cerebellum in the setting of lamin B2 deficiency. *Proc Natl Acad Sci U S A.* 2010;107(11):5076–81. 10.1073/pnas.0908790107 20145110 PMC2841930

[94] Frost B Bardai FH Feany MB: Lamin Dysfunction Mediates Neurodegeneration in Tauopathies. *Curr Biol.* 2016;26(1):129–36. 10.1016/j.cub.2015.11.039 26725200 PMC4713335

[95] Jung HJ Lee JM Yang SH: Nuclear lamins in the brain - new insights into function and regulation. *Mol Neurobiol.* 2013;47(1):290–301. 10.1007/s12035-012-8350-1 23065386 PMC3538886

[96] Kim Y Sharov AA McDole K: Mouse B-type lamins are required for proper organogenesis but not by embryonic stem cells. *Science.* 2011;334(6063):1706–10. 10.1126/science.1211222 22116031 PMC3306219

[97] Giacomini C Mahajani S Ruffilli R: Lamin B1 protein is required for dendrite development in primary mouse cortical neurons. *Mol Biol Cell.* 2016;27(1):35–47. 10.1091/mbc.E15-05-0307 26510501 PMC4694760

[98] Ast T Michaelis S Schuldiner M: The Protease Ste24 Clears Clogged Translocons. *Cell.* 2016;164(1–2):103–14. 10.1016/j.cell.2015.11.053 26771486 PMC4715265

